# Examining Intrapersonal and Social Environment Factors Associated with Condom Use and HIV Testing Among Young Adult MSM Living in Beirut, Lebanon

**DOI:** 10.1007/s10461-025-04943-0

**Published:** 2025-11-26

**Authors:** Jeffrey D. Grant, Harold D. Green, Matt Mutchler, Susan Kegeles, Elie Ballan, Glenn J. Wagner

**Affiliations:** 1 Department of Applied Health Science, Indiana University School of Public Health, Bloomington, IN USA; 2 Department of Health Science, California State University, Dominguez Hills, Carson, CA USA; 3 AIDS Project (APLA) Health & Wellness, Los Angeles, CA USA; 4 Division of Prevention Science, University of California, San Francisco, San Francisco, CA USA; 5 M-Coalition, Coalition of MSM and HIV activists in the MENA region, Beirut, Lebanon; 6 Department of Applied Health Science, Indiana University School of Public Health, 809 E. 9th St., Room 201, Bloomington, IN 47405 USA; 7 Health Unit, RAND Corporation, 1776 Main St, Santa Monica, CA 90407 USA

**Keywords:** Social Networks, Men who have sex with men, HIV Testing, Condom Use

## Abstract

Men who have sex with men (MSM) are disproportionately affected by HIV, particularly in urban Middle Eastern areas like Beirut, Lebanon. Understanding factors influencing HIV prevention, such as condom use and HIV testing is crucial. This study used the Information-Motivation-Behavioral Skills (IMB) Model determinants and explored social environment factors to assess their associations with condomless sex and HIV testing. Model fit was also examined to compare our IMB model alone versus an extended model incorporating social environment factors. A sample of 164 MSM (ages 18–29) in Beirut was analyzed using IMB determinants (e.g., HIV knowledge, motivation, condom use self-efficacy) and social environment determinants (e.g., safer sex peer norms, network engagement in HIV testing and risk discussions). Bivariate associations and multivariate regressions were conducted, and model fit was assessed using likelihood ratio testing. Findings showed that our IMB model alone better predicted condomless anal sex, whereas neither model adequately fit HIV testing outcomes. Within our IMB model, higher HIV knowledge was associated with increased odds of condomless sex, while greater condom use self-efficacy was linked to reduced odds. These results suggest that condom use and HIV testing are influenced by different factors. Interventions should not only increase HIV knowledge but also enhance self-efficacy to reduce condomless sex.

## Introduction

According to UNAIDS, the Middle East and North Africa (MENA) region has seen a rapid increase in HIV infections—approximately a 61% increase since 2010 [1]. These new infections are largely concentrated in marginalized groups such as sex workers, men who have sex with men (MSM), and individuals who inject drugs [1]. Existing data suggest that MSM living in Lebanon have one of the highest HIV prevalence rates in the region [2]. Compared to the national Lebanese prevalence rate of 0.1% among individuals between the ages of 15–49 [3], studies have found prevalence rates among MSM living in Lebanon ranging from 1.5% to 18.6% [4–7]—indicating a significant disparity.

Pre-exposure prophylaxis (i.e., PrEP) uptake is relatively limited in Lebanon likely due to societal stigma and cost [8]. In a recent surveillance survey, only about 34% of MSM were using PrEP [4]; therefore, condoms and HIV testing remain two prominent options for HIV prevention among MSM living in Lebanon. While condomless anal sex has decreased over the years [9], condom use is inconsistent among MSM. For example, only 63% of MSM reported condom use with their most recent casual partner [4]. In a sample of MSM living in Lebanon, Assi et al. found that most participants had multiple sexual partners within the past three months and infrequent condom use [5]. Increasing condom use with casual partners will likely reduce HIV prevalence rates among MSM living in Lebanon [10]. Understanding the factors that contribute to condom use (or lack thereof) is imperative to prevent HIV transmission.

Knowing one’s HIV status can also serve as a critical prevention measure [11]. There has been an upward trend in HIV testing as Wagner and colleagues found that MSM in 2017 were more likely to have ever been tested compared to MSM in 2012 [9]; however, there is limited data regarding the factors that predict a recent HIV test within the past six months. With a majority of MSM having multiple partners and practicing inconsistent condom use [5], frequent testing can be instrumental in preventing HIV infections. An examination of the factors that may influence or detract from HIV testing may allow interventions to provide a more thoughtful and effective approach.

Several studies have explored factors associated with condomless anal sex and HIV testing among MSM living in Lebanon [5, 7, 9, 12, 13]; however, researchers have often focused their attention on individual-level determinants or have focused on a limited set of determinants across varying levels (i.e., individual, interpersonal, and social environment), and without a strong theoretical underpinning. The Information-Motivation-Behavioral Skills (IMB) framework provides a foundation for understanding HIV prevention behaviors [14]. In particular, the model suggests that the more knowledge or information that one has regarding HIV, having greater motivation (either through personal motivation or through social network members), and a greater sense of self-efficacy will increase the likelihood of HIV prevention behaviors (i.e., condom use and HIV testing). Several studies have used the IMB framework finding knowledge, motivation, and behavioral skills to be significant determinants of HIV prevention behaviors [14–16]; however, there has been limited research using the IMB model as a framework for understanding condom use and HIV testing among MSM living in Lebanon.

While the IMB model does provide an examination of intrapersonal factors [14], there is limited integration of the broader social environment including social network factors [17, 18]. Studies have indicated that the social environment in which MSM are embedded are related to HIV prevention behaviors [19–23]. For example, Ghanem and colleagues found that MSM living in Lebanon who were more integrated into gay communities were more likely to have been tested for HIV; however, they were also more likely to have engaged in condomless anal sex [24]. Additionally, Schneider et al. found that Black MSM living in Chicago were more likely to engage in condomless anal sex if there was at least one network member who supports condomless anal sex [22]. A study of Chinese MSM found that individuals who had a greater proportion of MSM network members who had been tested for HIV were also more likely to have been tested and less likely to engage in condomless anal sex [23]. Examining social network factors can better serve the development of effective network-based interventions for MSM living in Beirut.

Given these current gaps, the objectives of this study were to identify intrapersonal and social environment factors that predict HIV testing and condom use among MSM living in Beirut, Lebanon. We used the IMB model as a grounding framework to identify core determinants (information, motivation, and behavioral skills) and then incorporated social environment factors such as one’s social network and peer social norms. This study also assessed if a framework that integrates social environment factors was more suitable than just a model that examines the IMB determinants.

## Methods

### Participant Recruitment and Study Design

This study used a cross-sectional research design using baseline survey data from a community-based HIV prevention and sexual health promotion intervention for young MSM living in Beirut, Lebanon (see [9, 24, 25]). Recruitment of the cohort took place between July 2016 and March 2017, primarily using peer-based chain referral methods. Other recruitment methods included flyers, social media postings, and word of mouth. These additional methods were implemented near the end of recruitment to help reach the desired target sample size. Eligibility criteria included being assigned male at birth, male-identified, aged 18 to 29 years old, fluent in English or Arabic, residing in greater Beirut, and having had oral and/or anal sex with a man within the past 12 months.

For the peer-based chain referral methods, recruitment began with a small number of eligible individuals designated as “seeds”. These seeds were identified through community organizations working with MSM populations and our community advisory board. These initial seeds were purposively selected as they were well-connected and representative of the diversity within the community. All participants—including seeds, the seeds’ direct recruits, and those recruited through flyers, postings, and word of mouth—received three coupons to recruit members of their social network, resulting in multiple waves of participants. Participants were instructed to give a coupon to eligible MSM peers interested in participating in the study and to inform the recruit to call the study coordinator for coupon verification, eligibility screening, verbal consent procedures, and interview scheduling. The survey interview was administered at the project office, by either an MSM or female interviewer, depending on the participant’s preference. Participants were compensated $40 for the interview, as well as $10 for each recruit (up to 3) who enrolled in the study. For seeds who recruited peers, the average number of recruits was 2.0 and the average length of a referral chain was 2.4 (minimum of 1 and a maximum of 8). A total of 226 participants were recruited; however, respondents who reported living with HIV (*n* = 8), individuals who did not have complete data on the social network measures (*n* = 52) and demographics (*n* = 2) were removed for analyses resulting in a final analytic sample of 164 respondents.

## Measures

The survey was administered in English or Arabic, depending on the preference of the respondent, using computer-assisted interview software. The survey was developed in English and translated into Arabic using standard translation and back translation methods. Participants were given the option of completing the survey on their own or having an interviewer administer the survey.

### Outcomes

#### Condomless Anal Sex

Any condomless anal sex in the past three months was measured by asking respondents how many times they engaged in both receptive and insertive anal intercourse over the past three months and how many of these acts involved condom use. Due to the small cell sizes of individuals who engaged only in insertive condomless anal sex (*n* = 20), individuals who engaged only in receptive condomless anal sex (*n* = 24), and individuals who engaged in both insertive and receptive condomless anal sex (*n* = 19), we decided to collapse these categories and create a single binary variable (yes or no) to indicate whether any condomless anal sex (receptive and/or insertive) was reported. Individuals who reported not having anal sex (receptive or insertive) in the past 3 months were coded as ‘no’ for condomless anal sex.

#### HIV Testing History

For recent history of HIV testing, respondents were asked if they have ever been tested for HIV, and if they have been tested, if they had been tested within the past 6 months (yes or no).

### Information-Motivation-Behavioral Skills Determinants

#### HIV Knowledge

Participants HIV knowledge was assessed using an 18-item scale of true-false statements [26]. Questions included knowledge regarding HIV testing (e.g., “Because of the ‘window period’, it is possible to get an HIV-negative test result and still have HIV”), HIV transmission (e.g., “You have to have sex with someone more than once to get HIV”), and condoms (e.g., “Condoms made of lambskin protects against the HIV virus”). Correct answers were summed for a total score ranging from 0 to 18. Individual questions where the participant responded “Don’t Know” or did not answer the question were scored as 0.

#### Motivation for Protective Health Behaviors

Motivation for protective health behaviors was assessed by creating a composite variable from three question items (“On a scale of 1–10, how important is it to you to take care of your health?”, “On a scale of 1–10 how important is it to you that you use condoms every time you have sex?”, and “On a scale of 1–10, how important is it to you that you get tested for HIV regularly?”). The average across the three questions was calculated. A higher score represents greater motivation for protective health behaviors.

#### Condom Use Self-efficacy

To assess condom use self-efficacy, six Likert-scaled questions adapted from the Condom Use Self-Efficacy Scale were used [27], which included “I am confident that I can tell a sex partner that I want to use condoms,” and “I am confident that I can use a condom even when I believe that my partner is HIV negative.” Response options ranged from 1 (strongly agree) to 5 (strongly disagree). A mean item score was calculated (after item scores were reversed). A higher score represents a greater self-efficacy.

### Social Environment Determinants

#### Safer Sex Peer Norms

Safer sex peer norms were measured using a nine-item Likert scale that included questions from the research of Choi et al. [28, 29] and additional items regarding substance use (e.g., “Most of my friends think you should avoid having sex when you are drunk.”). Response options ranged from 1 (strongly disagree) to 5 (strongly agree), and the mean item score was calculated, with higher scores representing stronger safer sex peer norms.

#### Judgmental Peer Communication About Sexuality

To assess judgmental communication regarding sexuality and sexual behavior from peers, an 11-item measure was used [30, 31]. Respondents were asked to rate how often (never, rarely, often, always) they had expressed or felt judgmental attitudes with and by their peers when communicating about sexual behavior [e.g., “I have called a friend ‘stupid’ or ‘dumb’ for having sex without a condom (even as a joke)”; “If I had sex without a condom, a friend would judge me for it”]. A mean item score was calculated with higher scores representing higher judgementalism.

#### Social Network Characteristics

To assess the respondent’s social network, we used a personal, egocentric network approach, which focuses on the network of ties that surround the respondent [32, 33]. Participants were asked to list 20 individuals in their social network (i.e., “alters”) who were also MSM and with whom they had been in communication with in the past 6 months (by phone, email, in person, etc.). Respondents were asked to start by naming individuals who were most important to them. These individuals could include kin (immediate and extended family), friends, people they work with, neighbors, or other community members. Research has demonstrated that eliciting 20 network members and the connections among them can reliably capture the variability in most network characteristics [34]. Respondents were asked to describe characteristics of each alter and aggregate proportions were calculated. For this analysis, we used the proportion of alters who were believed to had ever tested for HIV, the proportion of alters believed to use condoms mostly/always and never/rarely, and the proportion of alters whom the respondent had discussed HIV risk. To protect network members named, participants were instructed to provide aliases, initials, or first name of each network member. Network members named were further anonymized by removing the aliases, initials, or first names and assigning an alter ID number. Additionally, participants were asked to provide their perceptions or opinions of alters rather than providing confirmation of their alters’ behaviors.

### Covariates

Covariates included age (in years), education (no university education vs. at least some university), currently employed (Yes/No), being in a committed relationship (Yes/No), residency legal status (Yes/No). These covariates were selected as previous studies have indicated they are associated with condom use and HIV testing [6, 7, 13].

## Analytic Strategy

Bivariate analyses were conducted for each variable in association with outcome variables (t-tests and chi-squares). Multivariate logistic regressions were used to evaluate determinants for each outcome variable. More specifically, two models for each outcome variable were built. The first model included only the IMB determinants. The second model also included social environment determinants. Models were compared to see which had better fit using likelihood ratio testing. Variance inflation factors (VIFs) were assessed for multicollinearity. VIFs for all models were below 2 and below the recommended cutoff of 5, indicating a low likelihood of multicollinearity [35].

Missing items in two of our scales, the Judgmental Peer Communication (*n* = 10) scale and the Condom Use Self-Efficacy scale (*n* = 3), were handled using a half-rule imputation. In other words, when respondents had answers for half or more of the items within the scales, the mean of the observed items was calculated. Half-rule imputation has been used in similar populations [36]. All analyses were conducted in RStudio (version 2025.09.0 + 387) [37].

## Results

### Descriptives

The sample consisted of 164 respondents. The mean (SD) age was 23.6 (2.91) years old. A majority of respondents had some university education (87%) and were employed (60%). Of participants that were unemployed, 65% were current university students. Half of the respondents were current university students (50%). A quarter of respondents were in a relationship (25%). A majority had legal residency in Lebanon (88%). In terms of condomless anal sex in the past three months, 38% of participants reported condomless sex. More than half (54%) of participants reported being HIV tested within the past 6 months. See Table 1 for more details.

**Table 1:** Descriptive characteristics and bivariate associations between study variables and outcome measures (Any condomless anal Sex; HIV testing in past 6 Months)

Variable	Overall (*N* = all)	Any condomless anal sex (CAS)			HIV tested in past 6 months		
	*N* = 164^*1*^	No CAS *N* = 101^*1*^	Any CAS *N* = 63^*1*^	*p*-value^2^	Not Tested *N* = 76^*1*^	Tested *N* = 88^*1*^	*p*-value^2^
Model-tested variables							
HIV knowledge	12.74 (2.36)	12.47 (2.45)	13.19 (2.15)	**0.049**	12.57 (2.47)	12.90 (2.26)	0.374
Motivation for protective health	9.09 (1.10)	9.26 (0.91)	8.82 (1.31)	**0.021**	8.86 (1.25)	9.30 (0.92)	**0.012**
Condom self-efficacy	4.50 (0.64)	4.65 (0.48)	4.26 (0.77)	**< 0.001**	4.47 (0.68)	4.53 (0.60)	0.541
Safer sex peer norms	2.18 (0.60)	2.26 (0.67)	2.06 (0.46)	**0.031**	2.11 (0.57)	2.25 (0.62)	0.142
Judgmental peer communication	1.94 (0.57)	1.88 (0.51)	2.03 (0.65)	0.102	1.87 (0.57)	1.99 (0.57)	0.196
Prop. alters talk HIV risk	0.66 (0.32)	0.66 (0.33)	0.66 (0.32)	0.994	0.62 (0.33)	0.69 (0.32)	0.190
Prop. alters never/rarely use condoms	0.09 (0.18)	0.08 (0.17)	0.10 (0.19)	0.539	0.11 (0.19)	0.07 (0.16)	0.112
Prop. alters mostly/always use condoms	0.38 (0.31)	0.41 (0.32)	0.32 (0.29)	0.085	0.36 (0.31)	0.39 (0.31)	0.436
Prop. alters HIV tested	0.49 (0.32)	0.46 (0.32)	0.53 (0.31)	0.184	0.43 (0.32)	0.54 (0.31)	**0.016**
Covariates (included in models for adjustment)							
Age	23.62 (2.91)	23.68 (2.98)	23.51 (2.80)	0.704	23.96 (2.78)	23.32 (3.00)	0.157
Education level				0.2174			0.719
No university education	21 (13%)	16 (16%)	5 (7.9%)		11 (14%)	10 (11%)	
At least some university education	143 (87%)	85 (84%)	58 (92%)		65 (86%)	78 (89%)	
Employed				**0.025**			> 0.9
No	66 (40%)	48 (48%)	18 (29%)		31 (41%)	35 (40%)	
Yes	98 (60%)	53 (52%)	45 (71%)		45 (59%)	53 (60%)	
In a committed relationship				**< 0.001**			> 0.9
No	123 (75%)	86 (85%)	37 (59%)		57 (75%)	66 (75%)	
Yes	41 (25%)	15 (15%)	26 (41%)		19 (25%)	22 (25%)	
Residency legal status				> 0.9			0.734
No	19 (12%)	12 (12%)	7 (11%)		10 (13%)	9 (10%)	
Yes	145 (88%)	89 (88%)	56 (89%)		66 (87%)	79 (90%)	
Informational-only variables (descriptive only)							
Current university student							
No	82 (50%)	—	—	—	—	—	—
Yes	82 (50%)	—	—	—	—	—	—
Number of Social Network Members	13.68 (4.75)	—	—	—	—	—	—

^*1*^ Mean (SD); n (%); “—” indicates variables not included in inferential analyses

^*2*^ Welch Two Sample t-test; Pearson’s Chi-squared test; “—” indicates variables not included in inferential analyses

Bolded values are statistically significant at the p < .05 level

### Bivariate Results

For condomless anal sex in the past 3 months, significant associations were with HIV knowledge *(p* = 0.049), motivation for protective health behaviors *(p* = 0.021), condom use self-efficacy *(p* < 0.001), and peer sex social norms *(p =* 0.031).

For HIV testing in the past 6 months, the only significant associations were motivation for protective health behaviors *(p* = 0.012) and the proportion of network members who have been tested for HIV (*p* = 0.016) (see Table 1).

### Multivariate Results

For condomless anal sex, our IMB model indicated significant associations with HIV knowledge and condom use self-efficacy. For every one unit increase in HIV knowledge, the adjusted odds of condomless sex increased by a factor of 1.21 (*p* = 0.043, 95% CI [1.01–1.46]) when controlling for all other variables. In contrast, the adjusted odds of condomless sex decreased by 75% for every one unit increase in condom use self-efficacy scores (aOR = 0.25; *p* = 0.001, 95% CI [0.11–0.52]). When considering the full model with social environment determinants, HIV knowledge was only marginally significant (*p* = 0.069). Condom self-efficacy remained significant. Safer sex peer norms was also significant (*p* = 0.024). For every one unit increase in safer sex peer norms, the adjusted odds of condomless anal sex decreased by 60% (aOR = 0.40, 95% CI [0.18–0.87]).

When considering HIV testing, the only significant predictor in both the IMB model and the full model was motivation for protective behaviors (see Table 2).

**Table 2:** Multivariable Logistic Regression Models Examining Factors Associated with Condomless Anal Sex and HIV Testing in the Past 6 Months

	**Condomless Anal Sex (IMB)**			**Condomless Anal Sex (IMB+Social)**			**HIV Test (IMB)**			**HIV Test (IMB + Social)**		
*Variable*	*aOR*	*CI*	*p*	*aOR*	*CI*	*p*	*aOR*	*CI*	*p*	*aOR*	*CI*	*p*
HIV Knowledge	1.21	1.01 – 1.46	**0.043**	1.21	0.99 – 1.49	0.069	1.07	0.93 – 1.25	0.347	1.02	0.87 – 1.20	0.783
Motivation for Protective Health Behaviors	0.83	0.58 – 1.17	0.286	0.80	0.55 – 1.16	0.243	1.55	1.13 – 2.18	**0.008**	1.47	1.06 – 2.10	**0.025**
Condom Self-Efficacy	0.25	0.11 – 0.52	**0.001**	0.30	0.12 – 0.67	**0.005**	0.90	0.51 – 1.54	0.691	0.98	0.53 – 1.79	0.943
Safer Sex Peer Norms				0.40	0.18 – 0.87	**0.024**				1.20	0.65 – 2.25	0.555
Judgmental Peer Comm.				1.77	0.83 – 3.90	0.144				1.59	0.84 – 3.10	0.164
Prop. Alters Talk HIV Risk				0.56	0.14 – 2.19	0.406				1.36	0.43 – 4.31	0.602
Prop. Alters Never/Rarely Use Condoms				2.19	0.13 – 30.40	0.568				0.32	0.03 – 2.76	0.315
Prop. Alters Mostly/Always Use Condoms				1.02	0.22 – 4.55	0.979				0.93	0.27 – 3.19	0.911
Prop. Alters HIV Tested				2.47	0.56 – 11.44	0.237				2.55	0.79 – 8.53	0.122
Observations	164			164			164			164		

All models controlled for age, higher education, employment status, relationship status, and legal status

Bolded values are statistically significant at the p < .05 level

### Model Fit

For condomless anal sex, our IMB model fit the data better than a null model (*p* < 0.001); however, there was not a significant difference between the IMB model and the IMB model plus social environment determinants (*p* = 0.158) (see Table 3 for more details). For HIV testing, the IMB model was no better than a null model (*p* = 0.203). Additionally, our IMB model including the social environment determinants was also no better than the null model (*p* = 0.172) (see Table 4 for more details).

**Table 3:** Comparison of IMB Framework Model and Expanded Model for Condomless Anal Sex

Model	Deviance	*df*	AIC	Models Compared	Deviance Diff.	p-value	
Null (Intercept Only)	218.47	163	220.47				
^**1**^ **IMB Framework Model**	**165.24**	**155**	**183.24**	**Null & IMB**	**53.231**	**< 0.001**	
^1^IMB + Social Environment	155.94	149	185.94	IMB & IMB + Social Environment	9.295	0.158	

^1^Models controlled for age, education, employment status, relationship status, and legal status

Bolded values are statistically significant at the p < .05 level

**Table 4:** Comparison of IMB Framework Model and Expanded Model for HIV Testing within the Past 6 Months

Model	Deviance	*df*	AIC	Models Compared	Deviance Diff.	p-value	
Null (Intercept Only)	226.47	163	228.47				
^2^IMB Framework Model	215.50	155	233.5	Null & IMB	10.973	0.203	
^2^IMB + Social Environment	207.67	149	237.67	Null & IMB + Social Environment	18.808	0.172	

^2^Models controlled for age, education, employment status, relationship status, and legal status

### Supplemental Analysis

Due to the poor fit of both our IMB model and our IMB model with social environment determinants for HIV testing, we built an additional model that only included determinants that were significant in our bivariate tests at the p-value < 0.1 level. Predictors included motivation for protective health behaviors and proportion of network members who had ever been tested for HIV. When fitting this model to the data, motivation for protective health behaviors and network members who had ever been tested for HIV were both significantly associated with HIV testing. The odds of HIV testing increased by a factor of 1.49 for every one unit increase in the motivation for protective health behavior scores. Additionally, the odds of HIV testing increased by a factor of 3.17 for every one unit increase in the proportion of network members ever been tested for HIV (see Table 5 for more details). When we looked at model fit, this reduced model fit the data better than a null model (*p* = 0.0376 (see Table 6 for more details).

**Table 5:** Supplemental Multivariable Logistic Regression Model Examining Factors Associated with HIV Testing in the Past 6 Months

*Variable*	*aOR*	*CI*	*p*
Motivation for Protective Behaviors	1.49	1.10 – 2.08	**0.013**
Prop. Alters HIV Tested	3.17	1.14 – 9.13	**0.029**
Observations	164		

Model controlled for age, higher education, employment status, relationship status, and legal status

Bolded values are statistically significant at the p < .05 level

**Table 6:** Supplemental Model Fit for Reduced Model Examining Factors of HIV Testing within past 6 months

Model	Deviance	*df*	AIC	Models Compared	Deviance Diff.	p-value	
Null (Intercept Only)	226.47	163	228.47				
^3^**Reduced Model (Motivation & Proportion of Alters Tested)**	**211.59**	**156**	**227.59**	**Null & Reduced**	**14.879**	**0.038**	

^3^Model controlled for age, education, employment status, relationship status, and legal status

Bolded values are statistically significant at the p < .05 level

## Discussion

The purpose of this study was to identify the intrapersonal and social environment factors that predict condom use and HIV testing among MSM living in Beirut, Lebanon. Our analyses suggest that the IMB model provided a useful framework in identifying determinants of condom use. Our results highlight that condom use self-efficacy and HIV knowledge are associated with condom use; however, there was not an improved model fit when considering the broader social environment. Additionally, our results indicate that our IMB model nor our IMB model including the broader social environment factors were well suited for identifying predictors of HIV testing.

When looking at condom use specifically, condom use self-efficacy decreased the odds of condomless anal sex, which is consistent with previous studies [38, 39]. In our IMB model, which fit the data better for condomless anal sex compared to a model that also included social environment factors, HIV knowledge was positively associated with condomless anal sex—contradicting the theoretical framework. Other studies have reported either null or positive associations between HIV knowledge and condomless sex [40–42]. Perhaps as knowledge increases, individuals assess situational risk differently—leading to engagement in condomless sex. Ndugwa Kabwama and Berg-Beckoff [43] suggest that knowledgeable individuals may hold optimistic bias or risk denial beliefs, which may shape their engagement in condomless sex. Additionally, motivation for protective health behaviors was significant at the bivariate level but not in our multivariate model, suggesting it may play a smaller role in condom use decision making compared to HIV knowledge and condom use self-efficacy.

While our social environment model did not fit the data better, safer sex peer norms appear to play a role in condomless anal sex as safer sex peer norms were significant at both the bivariate and multivariate levels. Interventions may want to consider focusing on reshaping peer social norms rather than modifying one’s MSM social network environment, as the social network variables were not significant factors of condom use. Moreover, studies may want to consider how trust and closeness with sexual partners may influence condom use decision making. In a qualitative study, Goldenberg et al. found that MSM who perceived a greater degree of trust with their partners were more willing to forego condoms [44]. For MSM living in Beirut, it may be beneficial to explore what factors lead to increased trust particularly among casual partners and how these factors mediate the relationship between condom use self-efficacy and actual condom use.

When considering factors that influence HIV testing, motivation for protective health behavior was the only significant factor in our IMB model and our IMB model plus social environment determinants. Individuals who have higher motivation for protecting their health likely recognize that HIV testing is essential for overall health [45]. Instead of focusing on knowledge of HIV and condom self-efficacy as previous studies have done [9, 13], knowledge of HIV testing services and HIV testing self-efficacy would likely serve as better targets for intervention [46]. With that in mind, our IMB model and our IMB model with social environment determinants did not fit the data any better than a null model indicating that the current variable selection may not be suitable for assessing HIV testing behavior. The determinants that were chosen likely reflect Western ideas of HIV prevention behaviors, which may not be as applicable for MSM living in the Middle East [47]; therefore, a better understanding of what determinants influence HIV testing behaviors will be critical for interventions aimed at improving testing rates. As HIV testing requires interaction with healthcare providers, MSM may be uncomfortable or fearful due to potential MSM stigma [12, 48–50], likely leading to unmet testing needs [50, 51] or even falsified reports of HIV testing behavior. Future studies should explore MSM perceptions of MSM-based stigma and its relationship to HIV testing.

In our bivariate and supplemental analyses, we did find that individuals who had a greater proportion of network members who had been tested were also more likely to be tested. Social influence (e.g., encouragement to get tested) may be stronger among networks with a higher proportion engaging in HIV testing behavior [19]. Interventions may want to increase sexual health communication among MSM as these conversations may encourage and reinforce the need for testing [52, 53]. Additionally, interventions may want to consider integrating peer network change agents who can provide information and support to individuals who lack a network with high HIV testing behavioral norms [54].

There are limitations with this study. As the study design is cross-sectional, causal inferences cannot be made. Additionally, the sample predominately consisted of young, well-educated MSM; therefore, generalizations may not apply to a broader MSM population living in Beirut or MENA. The small sample size is also a limitation. A larger sample would likely provide more power to detect smaller effects; however, obtaining a larger sample in Beirut may be difficult due to the stigmatization of MSM [47]. Our recruitment efforts and sampling approach may have also led to sampling bias. For example, we only offered the survey in Arabic and English. With French being a prominent language, participation may have been affected without a French language survey option. Additionally, we used a peer-referral recruitment approach which may affect generalizability as individuals are recruiting network members who may share similar characteristics and behavioral practices; however, the peer-based recruitment approaches are common when recruiting hard to reach populations to assess sexual health [55, 56]. Due to small cell sizes, we used a variable for any condomless anal sex rather than examining models for receptive condomless anal sex and insertive condomless anal sex. Research among gay, bisexual and MSM living in Australia indicate different factors for receptive condomless anal sex compared to insertive condomless anal sex [57]. Future research should examine if similar patterns emerge among MSM living in Beirut. Furthermore, the data were collected from 2016 to 2017 and since then Lebanon has faced economic [58] and environmental disasters [59] as well as violent conflict [60]. Previous studies have indicated that violent conflict can significantly affect access to sexual health resources and lead to higher HIV rates [61]. Future studies should examine how this environmental context has affected MSM, their social networks, and their sexual health behaviors. For example, studies may want to assess how violent conflict may impact MSM mental health which may influence condom use and HIV testing [62]. Further, we did not assess how the complex religious and legal environment may also shape the ability for MSM to engage and receive sexual health care. While Lebanon does not explicitly criminalize same sex behavior [63, 64], law enforcement has used the Lebanese Penal Code Article 534 to target and arrest MSM [65]. This structural level stigma likely contributes to MSM being fearful of disclosing sexual behavior to healthcare providers and delaying necessary sexual healthcare [50]. Finally, our study did not test the IMB model directly and instead simply used it as a framework to see which determinants are associated with condomless anal sex and HIV testing. Due to the small sample size, we felt that a path analysis would be stymied; however, future studies should more adequately test the IMB model among MSM living in Lebanon.

Despite those limitations, the study does provide insights for intervention design. More specifically, we found that the IMB model determinants may be more suitable in the context of condom use behaviors than in the context of HIV testing. Additionally, our analyses highlight that HIV testing and condom use appear to be influenced by different factors, resulting in the need for different intervention approaches for each. HIV testing is an individual behavior conducted confidentially but often discussed broadly among sex partners and other MSM friends. Condom use is most often a dyadic behavior negotiated relationally but may be less often discussed among MSM friends. We found that motivations for protective health behaviors were more important for HIV testing than condom use. While knowledge can be powerful tool, interventions should not just consider increasing knowledge as a means to improving condom use and HIV testing; instead, interventions should consider building skills such as condom use self-efficacy.

While the social environment variables did not significantly contribute to our models (and in fact did not improve model fit), studies should further explore how MSM in Beirut interact with their social networks and the impact these network members have on their specific sexual health behaviors, separately. For example, studies may want to assess the frequency of sexual health communication with MSM peers to see if this influences condom use and/or HIV testing. More specifically, studies should assess the presence of network members who support condomless sex and the potential impact on condomless sex behavior [22].

## Conclusion

Our study illustrated that the IMB model can be helpful in identifying factors associated with condomless sex behaviors among younger MSM living in Beirut, Lebanon; however, it was not beneficial for identifying factors associated with HIV testing behaviors. As condomless anal sex and HIV testing have often been examined together, our study highlights that these two behaviors should be examined separately with unique determinants, as there were few commonalities between the two models.

## Data Availability

De-identified dataset is not available as participants did not consent to the use of data by researchers outside the study team.
